# A Novel Role for the Fifth Component of Complement (C5) in Cardiac Physiology

**DOI:** 10.1371/journal.pone.0022919

**Published:** 2011-08-01

**Authors:** Alaka Mullick, Jessy Tremblay, Zully Leon, Philippe Gros

**Affiliations:** 1 Biotechnology Research Institute, Montréal, Québec, Canada; 2 Microbiology and Immunology Department, University of Montréal, Montréal, Québec, Canada; 3 Biochemistry Department, McGill University, Montréal, Québec, Canada; Universität Würzburg, Germany

## Abstract

We have previously demonstrated that C5-deficient A/J and recombinant congenic BcA17 mice suffer from cardiac dysfunction when infected with *C. albicans* blastospores intravenously. During these studies we had observed that, even in the control un-infected state, BcA17 hearts displayed alterations in gene expression that have been associated with pathological cardiac hypertrophy in comparison to parental C5-sufficient C57Bl/6J (B6) mice. Of note was an increase in the expression of *Nppb*, a member of the fetal gene program and a decrease in the expression of *Rgs2*, an inhibitor of the hypertrophic response. We now report that C5-deletion has also affected the expression of other elements of the fetal gene program. Moreover deleting the C5a receptor, C5aR, has essentially the same effect as deleting C5, indicating a key role for C5a-C5aR signaling in the phenotype. Having noted a pathological phenotype in the un-infected state, we investigated the role of C5 in the response to cardiac stress. In previous studies, comparison of the expression profiles of *C. albicans*-infected BcA17 and similarly infected B6 hearts had revealed a paucity of cardioprotective genes in the C5-deficient heart. To determine whether this was also directly linked to C5-deficiency, we tested the expression of 5 such genes in the *C. albicans*-infected C5aR^−/−^ mice. We found again that deletion of C5aR recapitulated the alterations in stress response of BcA17. To determine whether our observations were relevant to other forms of cardiac injury, we tested the effect of C5-deficiency on the response to isoproterenol-induced hypertrophic stimulation. Consistent with our hypothesis, A/J, BcA17 and C5aR^−/−^ mice responded with higher levels of *Nppa* expression than B6 and BALB/c mice. In conclusion, our results suggest that an absence of functional C5a renders the heart in a state of distress, conferring a predisposition to cardiac dysfunction in the face of additional injury.

## Introduction

The innate immune system uses a number of surface and intracellular sensing molecules to detect the presence of invading microbes or products derived from them, triggering a so-called “pro-inflammatory” response [1]. The complement component C5 is cleaved to give rise to C5a, a potent pro-inflammatory molecule and C5b, that participates in the formation of the membrane attack complex (MAC). C5a is essential for the recruitment and activation of inflammatory cells such as granulocytes [2] and it mediates its effect primarily by binding a G-protein coupled receptor (GPCR), C5aR (or CD88) [2], [3]. Another C5a-binding receptor C5L2 (or GPR77) [4], has been described relatively recently. However, its role in C5a function is the subject of some debate [5]. The relevance of C5a to early pro-inflammatory response is highlighted by pathological situations, including sepsis, where damage to vital organs including the heart is driven in part by a cytokine storm, which includes excessive C5a production [6]. Also, in systemic lupus erythamatosis, C5a activation results in the disruption of the blood brain barrier integrity [7] and C5a-dependent activation of microglia and astrocytes has been proposed to contribute to progression of Alzheimer's disease [8]. Finally, C5a-mediated inflammatory response *in situ* has been shown to be an important pathological response during cerebral malaria [9], [10]. Hence, inhibition of C5a activity is an attractive strategy to treat or prevent a number of clinical conditions caused by excessive complement activation.

*Candida albicans* is an opportunistic pathogen that is part of the gut flora of most healthy individuals [11]. In the immuno-compromised host, *C. albicans* causes a wide spectrum of diseases ranging from superficial infections of the mucosa to life threatening disseminated disease [12]. Disseminated candidiasis, which is caused by deficiencies in the innate immune system, is characterized by fungal replication in vital organs such as the kidney, heart and brain, with the kidney being the most permissive site. Genetic analysis in inbred strains of mice has been used to investigate the major components of innate defenses whose impairment results in disseminated *C. albicans* infection [13], [14]. We have previously shown that a deficiency in the C5 component of complement is responsible for differential susceptibility of A/J (C5-deficient, susceptible) and C57BL/6J (C5-sufficient, resistant) mice to acute infection with *C. albicans* [14]. A/J mice have a 2-base pair deletion in the C5 gene as a result of which their serum lacks reactivity with anti-C5 antibody and consequently, any hemolytic activity [15]. Within 24 h of an intravenous challenge with 3×10^5^ *C. albicans* blastospores, A/J mice succumb to a dysregulated inflammatory response, necrotic damage of the heart, depressed cardiac metabolism and hypoglycemia. On the other hand, C5-sufficient C57BL/6J (B6) mice, suffer from renal insufficiency due to high fungal load and granulocyte infiltration of the kidneys over a protracted period of 7–21 days [16], [17]. The *C. albicans* susceptibility phenotype of A/J was recapitulated in the BcA17 mouse strain [18], a recombinant congenic line harboring 12% of the A/J genome (including the C5 mutation) fixed on a B6 resistant background [17].

Given the striking cardiac phenotype displayed by *C. albicans*-infected C5-deficient mice, we herein investigated the role of C5a-C5aR signaling in normal and stress-related cardiac gene expression. Our studies revealed an unexpected effect of C5-deficiency on cardiac physiology, including an altered gene expression pattern indicative of hypertrophy, and inability to respond appropriately to stress.

## Materials and Methods

### Mice

Eight to twelve week old, A/J, C57BL/6J, BALB/c and C5aR^−/−^ (C.129S4(B6)-C5aR1^tm1Cge^/J mice were purchased from The Jackson Laboratories (Bar Harbor, ME). To generate the C5aR^−/−^ mice, ES cells carrying the mutation were injected into C57BL/6 blastocysts, the chimeras were crossed to C57BL/6 females and the resulting heterozygote progeny were mated to C57BL/6 mice for 2 generations. At this point the mice were backcrossed 10 generations to BALB/c before being made homozygous. Therefore the effect of C5aR-deficiency has been evaluated by comparison of the C5aR^−/−^ phenotype with that of both B6 and BALB/c mice. The recombinant congenic strain BcA17 was purchased from Emerillon Therapeutics (Montreal, Québec, Canada). Mice were age and sex matched for all experiments. Housing and all experimental procedures were approved by the Biotechnology Research Institute Animal Care Committee, operating under the guidelines of the Canadian Council of Animal Care (Protocol numbers: 07-MAR-I-010, 08-MAR-I-010 and 09-MAR-I-010). Mice were monitored at regular intervals once the first clinical symptoms were observed to minimize their suffering by ensuring that the end point described in the protocol was respected.

### *C. albicans* infections

Candida albicans strain SC5314 was grown overnight in YPD medium at 30°C and harvested by centrifugation. The blastospores were washed twice in phosphate buffered saline (PBS) and re-suspended in it at the required density. For experimental infections, mice were injected via the tail vein with a 200 µl of suspension of 3×10^5^ C. albicans blastospores in PBS. Mice were closely monitored for clinical signs such as lethargy, loss of appetite, hunched back and ruffled fur. Mice exhibiting extreme lethargy were deemed moribund and were euthanized.

### Isoproterenol administration

Mice were injected sub-cutaneously with 10 µl/kg of a 10 mg/ml solution, resulting in a final dose of 100 mg/kg daily for 5 consecutive days. The injections were given at the same time (noon) each day and animals were euthanized 24 h after the last injection.

### Biochemical assays

The levels of creatine kinase (Pointe Scientific Inc., Canton, MI, USA) in the circulation were measured using a commercially available kit. To determine the levels of cytokines in the circulation, 12.5 µl of serum was analyzed using the BD^TM^ CBA Flex sets according to the manufacturer's instructions. Fluorescence levels were recorded using the BD^TM^ LSRII flow cytometry system (Becton-Dickinson Biosciences, CA, USA) using BD FACSDivaÿ acquisition software and the data analysis was carried out using the FCAP Array software.

### Semi-quantitative RT-PCR

Transcript levels were measured by semi-quantitative RT-PCR in a LightCycler (Roche Diagnostics, Laval, Québec) with the DNA SYBR Green I reaction (Roche Diagnostics). cDNA was synthesized from 1 µg of RNA with SuperscriptII reverse transcriptase (Gibco-Invitrogen, Burlington, Ontario, Canada), according to the instructions of the manufacturer. The sequences of the primers used for PCR amplification are described in Table 1. The *S-29* gene was used as a reference gene in PCR experiments [17]. As described in the Roche Applied Science Technical note LC16/2005, quantitation of test gene expression was performed by comparing the threshold cycle values. Thus comparative levels of RNA-X in sample A with reference to sample B =  (threshold cycle for X in A/threshold cycle for *S29* in A*)*/(threshold cycle for X in B/threshold cycle for *S29* in B).

**Table 1:** Sequence of primers for semi-quantitative RT-PCR.

Gene	Forward	Reverse
*Nppb*	5′ GAG GTC ACT CCT ATC CTC TGG 3′	5′ CC ATT TCC TCC GAC TTT TCT C 3′
*Myh7*	5′ ACT GTC AAC ACT AAG AGG GTC A 3′	5′ TTG GAT GAT TTG ATC TTC CAG GG 3′
*Myh6*	5′ CAG AGG AGA AGG CTG GTG TC 3′	5′ CGA ACA TGT GGT GGT TGA AG 3′
*Nppa*	5′ TGA AAA GCA AAC TGA GGG CT 3′	5′ CAG AGT GGG AGA GGC AAG AC 3′
*Acta1*	5′ GCA TGC AGA AGG AGA TCA CA 3′	5′ ATT TCC TTT CCA CAG GGC TT 3′
*S29*	5′ GTC TGA TCC GCA AAT ACG GG 3′	5′ AGC CTA TGT CCT TCG CGT ACT 3′
*Rgs2*	5′ AGG ATT GGA AGA CCC GTT TGA GC 3′	5′ CAT CAA ATG CTT CTG CCC AGA GC 3′
*Calr*	5′ CCT GCC ATC TAT TTC AAA GAG CA 3′	5′ GCA TCT TGG CTT GTC TGC AA 3′
*Sepinh1*	5′ GCC GA 5′ GCC GAG GTG AAG AAA CCC C 3′	5′ CAT CGC CTG ATA TAG GCT GAA G 3′
*Gadd45*	5′ GGG AAA GCA CTG CAC GAA CT 3′	5′ AGC ACG CAA AAG GTC ACA TTG 3′
*Nrp1*	5′ GAC AAA TGT GGC GGG ACC ATA 3′	5′ TGG ATT AGC CAT TCA CAC TTC TC 3′

### Statistical analysis

**Figure 1:**
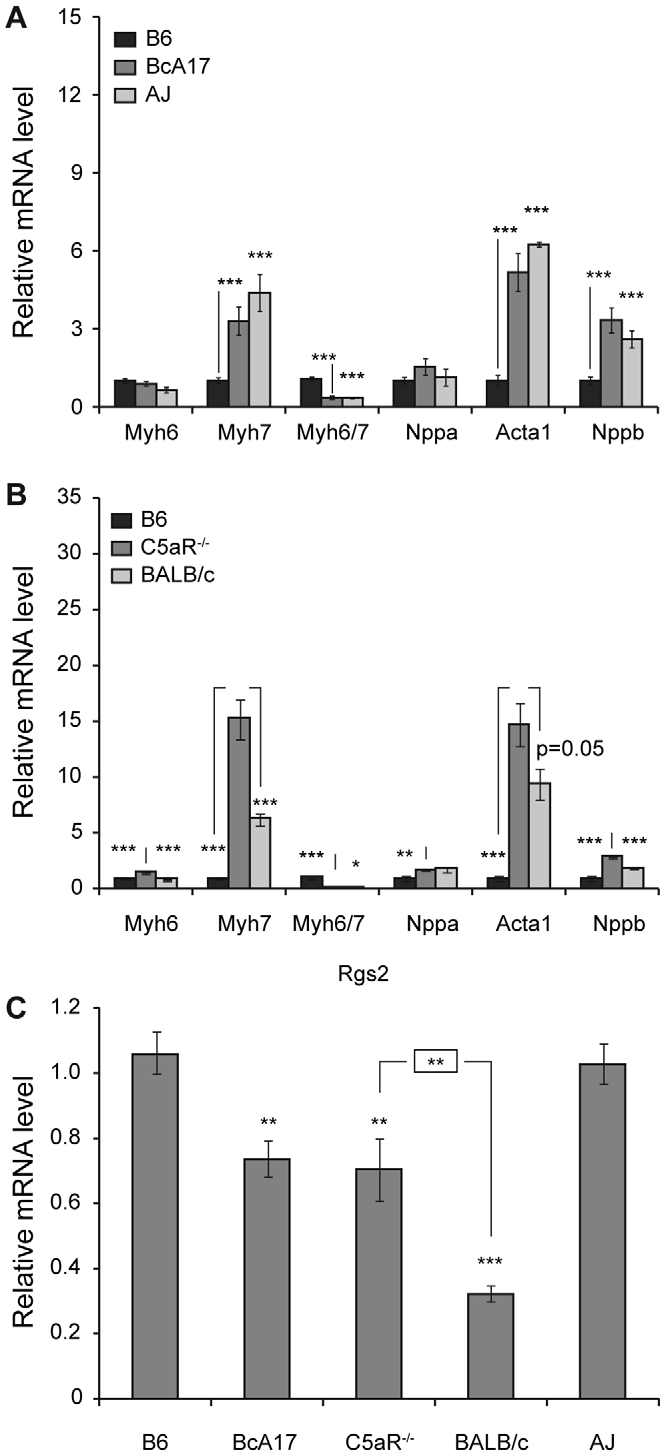
Effect of C5-deficiency on the hypertrophic gene expression program. Semi-quantitative RT-PCR was used to determine the levels of the indicated genes in heart RNA from C57Bl/6J (B6 n = 13), BcA17 (n = 13), A/J (n = 12) (**A**), B6 (n = 9), C5aR^−/−^ (n = 9) and BALB/c (n = 6) (**B**), and B6 (n = 11), BcA17 (n = 8), and C5aR^−/−^ (n = 6), BALB/c (n = 6) and A/J (n = 17) (**C**). In all panels transcript levels are presented as relative amounts with respect to those in control B6 RNA. The figure represents data from at least two independent experiments. The error bars indicate the standard errors of the means. Statistically significant differences with control B6 (A and C), C5aR^−/−^ (B) or BALB/c (C: boxed) values, are indicated by asterisks (* p<0.05, **p<0.01 and *** p<0.001).

**Figure 2:**
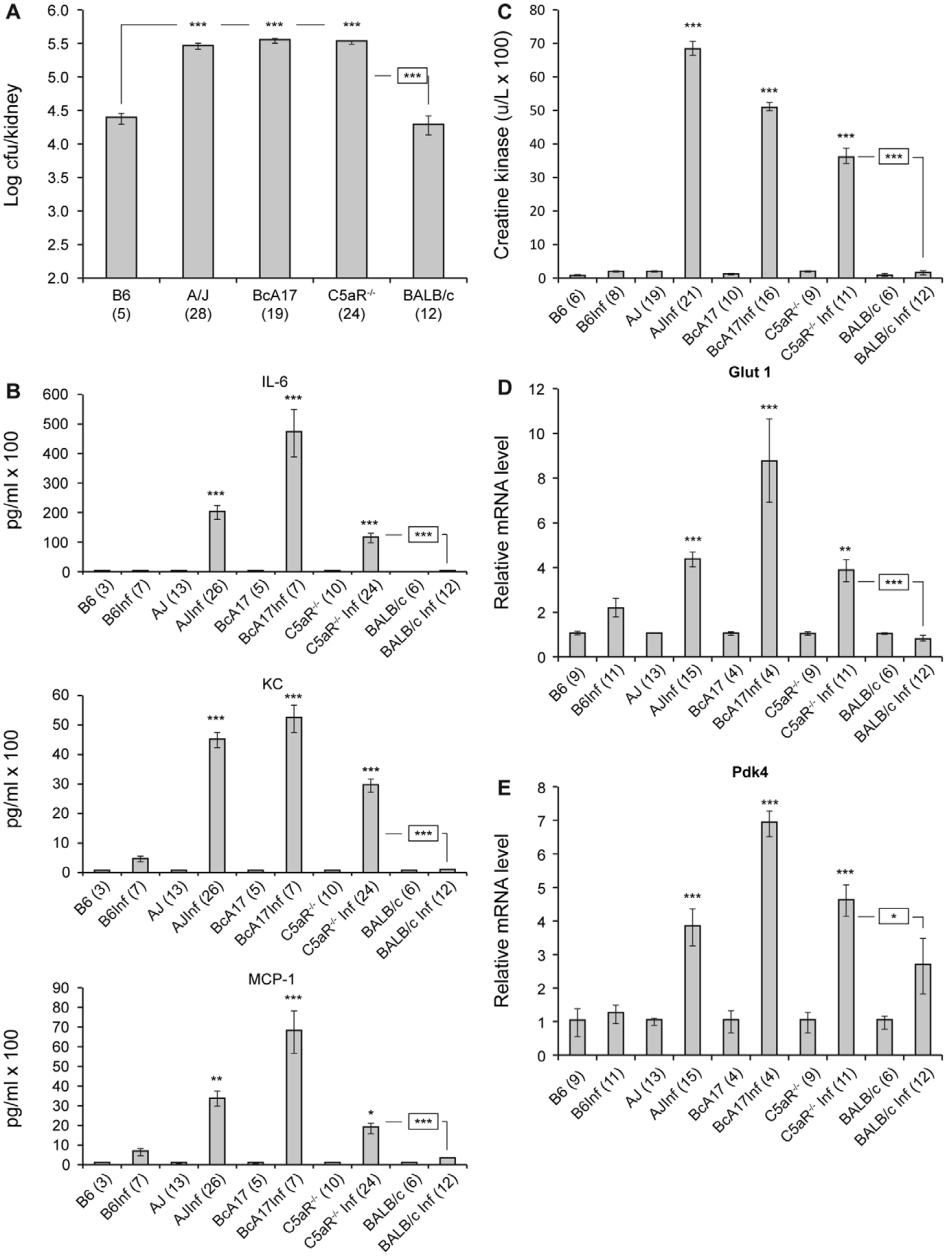
*C. albicans* infection in C5aR^−/−^ mice. Control and *C. albicans*-infected B6, A/J, BcA17, C5aR^−/−^ and BALB/c mice were euthanized 24 h post-infection. Kidneys were used for fungal load determination (**A**). Blood was collected to measure the inflammatory response (**B**) and creatine kinase (**C)**. Semi-quantitative RT-PCR was used to determine the relative level of expression of *Glut1* (**D** *)* and *Pdk4* (**E** *)*. The figure represents data from at least two independent experiments. The numbers in brackets correspond to the number of mice used to generate the figure. The error bars indicate the standard errors of the means. Statistically significant differences with *Candida*-infected B6 or BALB/c (boxed) values are indicated by asterisks (* p<0.05, **p<0.01 and *** p<0.001).

**Figure 3:**
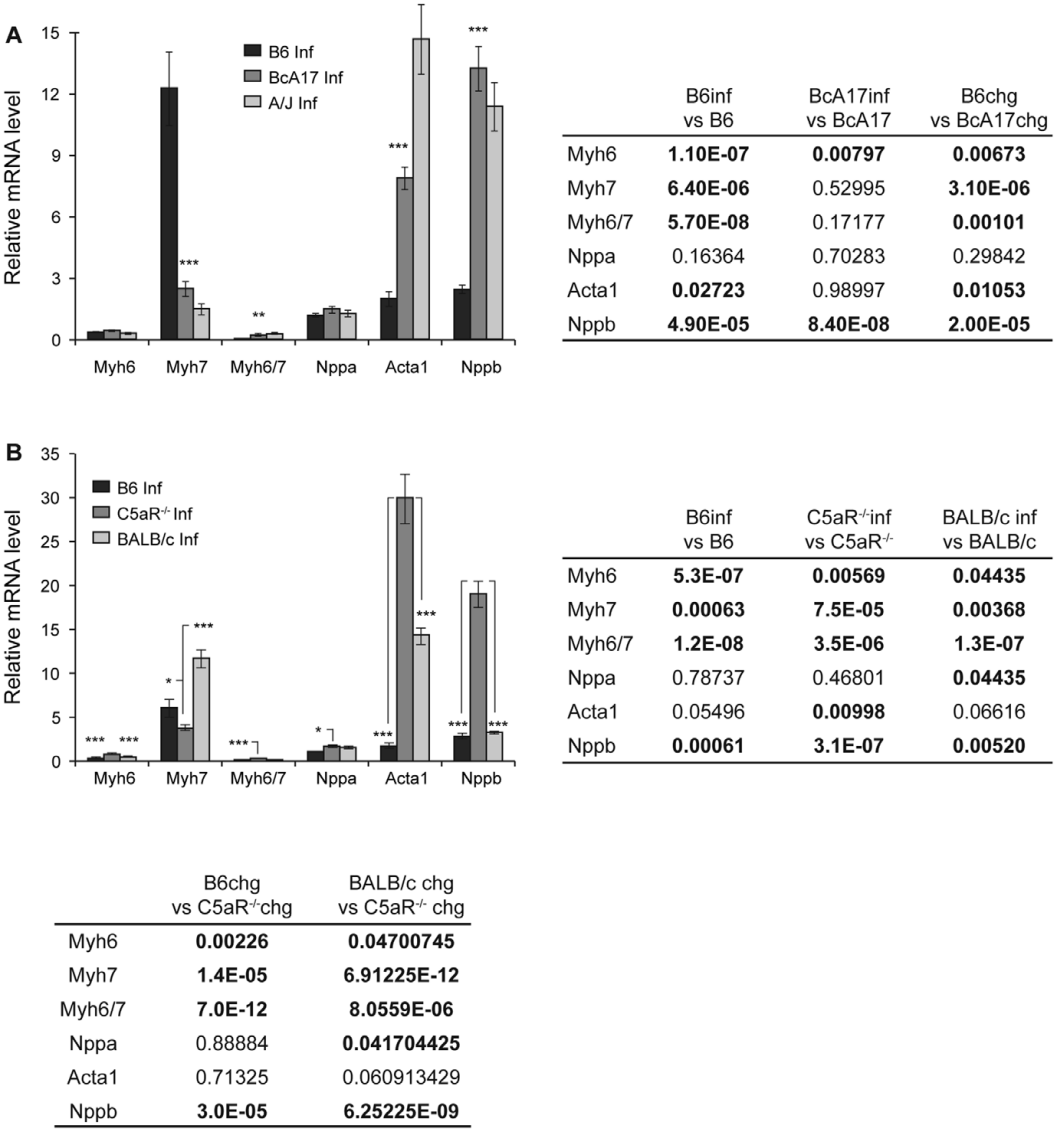
The effect of C5-deficiency on expression of hypertrophic gene expression program in response to *C. albicans* infection. Semi-quantitative RT-PCR was used to determine the levels of the indicated genes in heart RNA from *C. albicans*-infected B6, BcA17, A/J, C5aR^−/−^ and BALB/c mice (B6 inf, BcA17 inf, A/J inf (**A**) and B6 inf, C5aR^−/−^ inf, and BALB/c inf (**B**). Transcript levels in the infected (A: B6 n = 10, BcA17 n = 11, A/J n = 12; B: B6 n = 11, C5aR^−/−^ n = 11, BALB/c n = 12) hearts were calculated relative to those in un-infected B6. The figure represents data from at least two independent experiments. The error bars indicate the standard errors of the means. Statistically significant differences with *Candida*-infected B6 (A) or C5aR^−/−^ (B) hearts are indicated by asterisks (* p<0.05, **p<0.01 and *** p<0.001). P-values for the change in gene expression upon infection in a given strain (Strain Inf vs Strain) or between strains (Strain 1 chg vs Strain 2 chg) is tabulated with statistically significant changes in bold.

**Figure 4:**
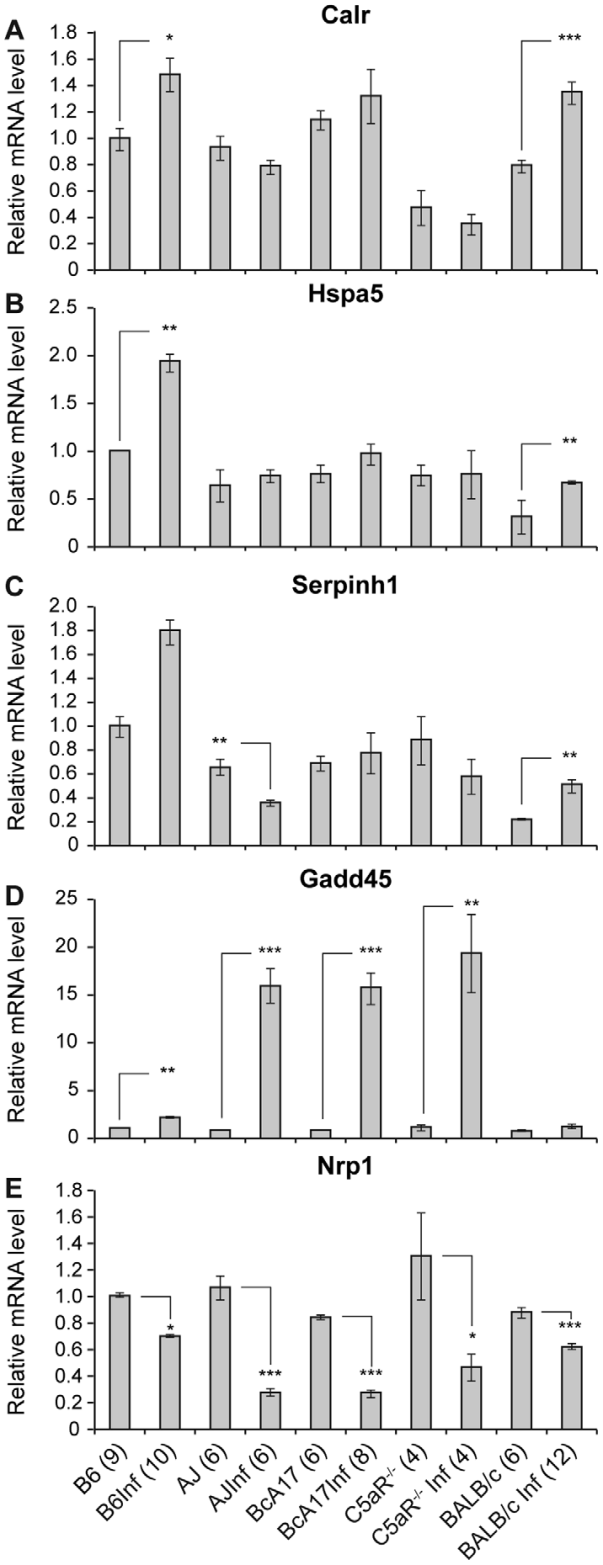
The effect of C5-deficiency on expression of stress response genes in response to *C. albicans* infection. Semi-quantitative RT-PCR was used to determine the levels of the indicated genes in heart RNA from control and *C. albicans*-infected B6, A/J, BcA17, C5aR^−/−^ and BALB/c mice. Transcript levels in the control and infected hearts were calculated relative to those in un-infected B6. The numbers in bracket refer to the number of mice used to generate the figure. The figure represents data from at least two independent experiments. The error bars indicate the standard errors of the means. Statistically significant differences in infected hearts with respect to their controls are indicated by asterisks (* p<0.05, **p<0.01 and *** p<0.001).

**Figure 5:**
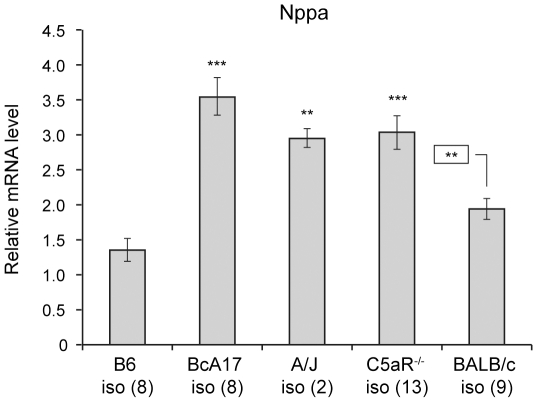
The role of C5-deficiency on the response to isoproterenol administration. Semi-quantitative RT-PCR was used to determine the levels of *Nppa* in heart RNA from control and isoproterenol-treated B6, A/J, BcA17, C5aR^−/−^ and BALB/c mice. Transcript levels in the treated hearts were calculated relative to those in un-treated B6. The figure represents data from at least two independent experiments. The error bars indicate the standard errors of the means. Statistically significant differences with isoproterenol-treated B6 or BALB/c (boxed) hearts are indicated by asterisks (* p<0.05, **p<0.01 and *** p<0.001).

The statistical significance of the difference observed for relative RNA expression levels (Figs. 1, 2D, 2E, 3, 4 and 5), fungal load (Fig. 2A), cytokine levels (Fig. 2B) and creatine kinase levels (Fig. 2C) between experimental and control samples was assessed using a 2-tailed Students t-test assuming equal variance in the two data sets.

## Results

### Cardiac hypertrophic gene expression in C5-sufficient and deficient mice

In previous studies, transcript profiling experiments using heart RNA showed that, in addition to a large set of genes whose expression was altered in response to *C. albicans* infection, a subset of genes was differentially expressed in a C5-dependent fashion, and this, prior to infection [17] (NCBI GEO accession number GSE3381). Notable amongst these was *Nppb (*B-type natriuretic peptide; BNP), a member of the fetal gene program and marker of pathological hypertrophy [19], and *Rgs2* (Regulators of G-protein Signaling; RGS) an inhibitor of hypertrophy [20], suggesting that C5 deficiency may have intrinsic consequences on cardiac structure and function.

We therefore decided to extend our studies to other elements of the fetal gene program associated with maladaptive hypertrophy. In addition, as a first step towards elucidating the mechanism of the C5-effect, we examined the effect of deleting the major receptor for C5a, (C5aR), on the hypertrophic expression profile. Semi-quantitative RT-PCR was used to compare the cardiac expression levels of the fetal gene program [α-myosin heavy chain *(Myh6)*, ß-myosin heavy chain *(Myh7)*, the *Myh6/Myh7* ratio, A-type natriuretic peptide (*Nppa)*, alpha skeletal actin (*Acta*), B-type natriuretic peptide (*Nppb*)] in BcA17, A/J, and C5aR^−/−^ with expression levels in C5-sufficient B6 and BALB/c mice (Fig. 1 A and B). In support of the idea of cardiac stress in the absence of functional C5, regardless of whether it was due to C5 (BcA17: Fig. 1A) or C5aR (C5aR^−/−^ : Fig. 1B) deletion, the levels of *Myh7,* and *Nppb* were higher (p<0.001) and the *Myh6/Myh7* ratio was lower (p<0.001 (BcA17 vs B6, C5aR^−/−^ vs B6) and p<0.05 (C5aR^−/−^ vs BALB/c) in the absence of C5a activity. *Acta1* expression was also similarly affected by the absence of functional C5 (BcA17 vs B6 p<0.001 and C5aR^−/−^ vs B6 p<0.001 and p = 0.0501 for the C5aR^−/−^- BALB/c comparison). It is notable that BcA17 shared the hypertrophic gene expression pattern with A/J, its C5-deficient parental strain and that C5aR deletion results in similar effects on cardiac gene expression, despite being on a distinct C5-sufficient background, that of BALB/c (B6 vs BALB/c p<0.001 for *Nppb*, *Acta1, Myh7* and the *Myh6*/*Myh7* ratio and p<0.05 for *Nppa*). Equally relevant to cardiac hypertrophy was the reduced expression of *Rgs2* in un-infected BcA17 when compared to the C5-sufficient B6 hearts (Fig. 1C p<0.01). Deleting C5aR also had an impact on cardiac *Rgs2* expression, since the levels in C5aR^−/−^ mice are significantly different from both B6 and BALB/c parental strains, being lower than B6 (p<0.01), but higher than BALB/c (p<0.01). Taken together, our data suggested a link between C5-deficiency and cardiac hypertrophy and hence merited further investigation.

### Response of C5aR^−/−^ mice to *C. albicans* infection

To determine whether the changes in cardiac gene expression in C5aR^−/−^ mice were also associated with the differential susceptibility phenotype of A/J and BcA17 vs. B6 to *C. albicans* infection, we investigated the response of the C5aR^−/−^ mice to *Candida* infection. Therefore, A/J, BcA17, C5aR^−/−^, BALB/c and B6 mice were administered 3×10^5^ *C. albicans* intravenously. As described in Mullick *et al*. [17], twenty-four hours post-infection, the C5-deficient A/J and BcA17 mice were moribund whereas the B6 and BALB/c mice showed no clinical symptoms. At this point C5aR^−/−^ mice also displayed severe symptoms of disease progression (lethargy, ruffled fur and hunched back). All *C. albicans*-infected mice and un-infected controls were euthanized and kidneys were harvested to evaluate fungal growth *in vivo*, since they provide the most permissive site for *C. albicans* replication. Hearts and blood samples were collected for biochemical analysis. Figure 2 shows that, in terms of kidney fungal load (p<0.001), the inflammatory response (IL6 p<0.001, KC p<0.001 and MCP-1 p<0.001 (C5aR^−/−^ vs BALB/c) and p<0.05 (C5aR^−/−^ vs B6)) and necrotic damage to the heart (p<0.001) *C. albicans*-infected C5aR^−/−^ mice were significantly different from their C5-sufficient counterparts (B6 and BALB/c), indicating that the C5-deficiency phenotype is a consequence of a lack of C5a-C5aR signaling. Finally, given that our previous studies indicated that *Candida-*infected C5-deficient mice suffer from altered cardiac metabolism in comparison to the C5-sufficient B6 mice, we measured cardiac RNA levels of *Glut1* (Fig. 2D) and *Pdk4* (Fig. 2E). Consistent with our earlier reports, levels of *Glut1* and *Pdk4* are higher in *C. albicans*-infected C5aR^−/−^ hearts than in similarly infected B6 (*Glut1* p<0.01, *Pdk4* p<0.001) and BALB/c (*Glut1* p<0.001, *Pdk4* p<0.05) hearts respectively. Our data clearly demonstrate that fungal load, the dysregulated inflammatory response, necrotic damage to the heart and changes in cardiac glucose oxidation upon *C. albicans* infection, are indeed linked to the C5 status and C5aR mediates the effect.

### Changes in cardiac fetal gene expression upon *C. albicans* infection

Given that the reactivation of the fetal gene program is indicative of cardiac pathology, we wanted to determine whether it was further modulated by *C. albicans* infection in a C5-dependent manner. Therefore semi-quantitative RT-PCR was carried out using heart RNA of control and infected mice (24 hrs post-infection). Fig. 3 shows the level of expression of the different genes after infection, relative to that in control B6 hearts. As shown in the figure, *Myh7* is induced significantly only in B6 and BALB/c mice (Figs. 2A and 3A: *Myh7* induced 12-fold in B6 p<0.001; while there was no change in BcA17; Figs. 2B and 3B: *Myh7* increased 6.22-fold in B6 p<0.001 and 1.77-fold in BALB/c p<0.01, but reduced 3.84-fold in C5aR^−/−^ p<0.001) resulting in higher induced levels of *Myh7* in *Candida*-infected B6 and BALB/c hearts compared to similarly infected BcA17 and C5aR^−/−^ hearts (Fig 3A and B). Also, as a consequence of changes in *Myh7* levels, the *Myh6/Myh7* ratio changed from being higher in the uninfected state to lower in the infected C5-sufficient hearts (Fig 3A and B). In contrast to the expression of *Myh7*, which is more efficiently increased in response to infection in the C5-sufficient state, the expression of *Nppb* was not only higher in the un-infected state (Fig 1), but was induced more strongly upon infection, in the absence of functional C5 (Fig. 3A: 5-fold in BcA17 vs. 3-fold in B6 p<0.001 and Fig. 3B: 6.6-fold in C5aR^−/−^ vs 2.75-fold in B6 p<0.001 and 1.33-fold in BALB/c p<0.001), resulting in further exacerbation of the C5-dependent differences in *Nppb* expression. Although it is not clear why some genes are up-regulated and others down-regulated, our results indicate that the manner in which their expression will change is largely dependent on the C5-status, highlighting the importance of C5 in the response of the heart to an insult such as infection by *C. albicans.*

### Changes in cardiac stress response upon *C. albicans* infection

Comparison of cardiac gene expression profiles of *C. albicans*-infected BcA17 vs B6 mice [17] suggested a deleterious effect of C5-deficiency on the stress response of the heart. Therefore we chose 5 of the differentially expressed genes with a role in the response to injury, and examined their expression during *C. albicans* infection in A/J, BcA17, C5aR^−/−^, B6 and BALB/c mice. Fig. 4 shows that *Candida*-infected B6 and BALB/c hearts up-regulate the *Calr* (B6: p<0.05; BALB/c: p<0.001), *Hspa5* (B6: p<0.01; BALB/c: p<0.01) and *Serpinh1* (B6: p = 0.057; BALB/c: p<0.01) expression upon infection, whereas the C5aR^−/−^ and C5-deficient strains do not. Although all strains up-regulate GADD45 the fold change in A/J, BcA17 (19-fold p<0.001) and C5aR^−/−^ (17-fold p<0.001) mice is significantly higher than that in B6 (2- fold p<0.01) and BALB/c (2.3-fold) (B6 vs BcA17 p<0.01; B6 vs C5aR^−/−^ p<0.001; BALB/c vs C5aR^−/−^ p<0.001). Similarly although all strains down-regulate *Nrp1*, the fold change is higher in the BcA17 (3.97-fold p<0.001) and C5aR^−/−^ (2.87-fold p<0.05) vs 1.44-fold in B6 (p<0.05) and BALB/c (p<0.001)(B6 vs BcA17 p<0.05; B6 vs C5aR^−/−^ p<0.05; BALB/c vs C5aR^−/−^ p<0.001). Therefore our data reveals a fundamental alteration in the response of the heart to stress in the absence of functional C5.

### Response to isoproterenol

To determine whether the deleterious effect of C5-deficiency applies to other forms of cardiac injury, and to minimize the complications inherent to an infectious process, we tested the response of the A/J, BcA17, C5aR^−/−^, BALB/c and B6 mice to a sub cutaneous injection of 100 mg/kg isoproterenol for 5 consecutive days. 6 out of 8 A/J and 1 out of 8 BALB/c mice succumbed to isoproterenol treatment on days 4 and 5 of treatment. However, BcA17 and C5aR^−/−^ mice did not exhibit clinical symptoms. In addition, other changes observed upon *C. albicans* infection, such as inflammatory activity, creatine kinase levels and cardiac glucose metabolism were not significantly affected by isoproterenol administration (data not shown).

To further examine the response to isoproterenol, we measured the changes in *Nppa,* a known response to this treatment [21]. Figure 5 shows that isoproterenol-treated hearts of BcA17 and C5aR^−/−^ mice express significantly higher levels of *Nppa* than the hearts of similarly treated B6 (BcA17 vs B6 p<0.001; C5aR^−/−^ vs B6 p<0.001) and BALB/c mice (C5aR^−/−^ vs BALB/c p<0.01), amplifying the difference already present in the control hearts (Fig. 1). Therefore the exquisite cardiac sensitivity of C5-deficient hearts is not limited to *C. albicans* infection and includes adrenergic stimuli such as isoproterenol.

## Discussion

Our previous studies had suggested that C5-deficiency might predispose mice to cardiac dysfunction since they suffer from depressed cardiac metabolism within 24 h of infection with *C. albicans* blastospores [17]. Analysis of cardiac gene expression profiles had indicated an inability of the BcA17 mice to mount an appropriate stress response in comparison to B6, the C5-sufficient parental strain. Moreover, even in the normal uninfected state, the C5-deficient heart expressed higher levels of *Nppb*, signifying a deviation from normal cardiac physiology. We now report that the expression of other elements of the so-called fetal gene program (*Myh, Nppa* and *Acta1*), is also affected by C5-deficiency. Although, it is not always possible to make a direct correlation between the expression of the so-called “fetal gene program” and cardiac dysfunction, it is clear that the reactivation of these fetal genes is a sign of cardiac stress/pathology, since physiological hypertrophy does not induce their expression. Indeed, ANP and BNP may be more reliable as markers for cardiac stress/pathology rather than for hypertrophy [19]. In support of this view, a comparison of gene expression patterns in adult, fetal and failing hearts reveals a striking similarity in the pathways activated in fetal and failing hearts [22]. Moreover physiological hypertrophy does not induce such a reactivation [23]. Another revelation of the microarray analysis was the lower levels of the transcript for *RGS2*, a negative regulator of Gαq signaling in BcA17 hearts compared to B6 hearts [20]. A decrease in RGS2 levels is of special interest to this study since it has been shown that such a decrease can result in an exaggerated response to the stress of pressure overload, characterized by hypertrophy and heart failure, in comparison to wild-type controls [24]. In addition *Rgs2*, but not *Rgs3–5* are down-regulated in murine models of hypertrophy [25]. However, it is important to note that, in contrast to the fetal gene program, RGS2 is not a general marker for hypertrophy. In the heart, it's expression is increased only upon G protein-mediated hypertrophic signaling [21]. We have shown that C5a-deficiency on a B6 background (BcA17) results in a decrease in *Rgs2* expression. Curiously, A/J, the C5-deficient parent of BcA17 mice does not display this decrease, suggesting that this phenotype is not inherited from this parent. The *Rgs2* locus maps to chromosome 1 at 67 cM and in BcA17 mice, this chromosomal region is derived from the B6 parent according to the mapping of microsatellite markers: BcA17 (described as BcA70 in [18]) genomic DNA carries B6 alleles for 17 markers between D1Mit386 (59.5 cM) to D1Mit425 (81.60 cM) including D1Mit218 at 67 cM. Thus changes in *Rgs2* expression in BcA17 with respect to the parental B6, have probably developed as an adaptation of C5-deficiency on the B6 background. At this point it is unclear whether RGS2 function is normal in the A/J mice, since we have only examined transcript levels. However, it has been reported that A/J mice also suffer from aberrant regulation of GPCR signaling, since, in contrast to B6 mice, they failed to desensitize ß-adrenergic receptors and down-regulate adenylyl cyclase activity upon isoproterenol administration [26]. Further studies will be required to determine the association of deviant ß-AR signaling in A/J mice with C5-deficiency.

Similarly, our observation that *Rgs2* expression in BALB/c hearts was significantly lower (32%) than in the hearts of the other C5-sufficient strain, B6, was surprising and indicated that one or more C5-independent factor(s) were impinging upon cardiac function in BALB/c mice. Indeed, BALB/c mice are known to succumb to spontaneous heart failure as a result of dystrophic cardiac calcinosis (DCC), a degenerative condition characterized by abnormal calcium deposition and necrotic foci in the heart [27], [28]. Although biochemical changes underlying this phenotype have not been extensively investigated in BALB/c mice themselves, a similar condition has been studied in mice expressing a mutant form of desmoglein 2 (lacking the extracellular adhesive domain), that develop cardiac fibrosis and calcinosis [29]. In these mice cardiac levels of *Myh7* and *Nppa* were higher in mutant versus wild-type mice, wherein wild-type mice have a mixed genetic background, including that of B6. Our data has also revealed higher levels of these cardiac stress markers in BALB/c hearts compared to B6 hearts (Fig. 1B *Myh7:* BALB/c vs B6 6.27-fold p<0.001; *Nppa*: BALB/c vs B6 1.84-fold p<0.05). Moreover, the desmoglein 2 mutant mice express lower levels of *Rgs2* than their wild-type counterparts, by 8 weeks of age. Therefore it is likely that the low *Rgs2* (Fig. 1C) levels and the relatively severe reaction of BALB/c mice to isoproterenol administration (1 out of 8 mice did not survive) are a consequence of the DCC phenotype. It is of note that the C5aR^−/−^ mice express significantly higher levels of *Rgs*2 and their response to isoproterenol was distinct from that of BALB/c mice, despite sharing most elements of their genetic background with this parent. However since the levels are lower than that in B6 hearts (Fig. 1C), one possible explanation is that, early in the breeding scheme, when the mutation was on a B6 background, *Rgs2* expression was reduced as an adaptation to C5aR-deficiency on a B6 background.

Our observation that the C5aR^−/−^ mice recapitulate the cardiac phenotype characterized by reactivation of the fetal gene expression, *Rgs2* down-regulation and sensitivity to *Candida* infection and isoproterenol administration, reveals the first step in the mechanism of the C5 effect. Although C5 is cleaved to give rise to two peptides, C5a and C5b, our data signifies the importance of C5a in this phenotype. Moreover C5a can mediate its effect via C5aR or C5L2 [2], but we have shown that the majority of the effects are recapitulated by C5aR deletion and therefore, if C5L2 plays a role, it is relatively minor or redundant. Thus the first important finding of our study is that in the absence of C5a-C5aR signaling, the heart displays signs of maladaptive hypertrophy or stress. Moreover, given the wide range of cell types that express these receptors [5], it is likely that the role of C5 in normal physiology is more significant than earlier appreciated.

Having observed an effect of C5-deficiency in the normal state, we examined the consequences of cardiac injury in the absence of functional C5a-C5aR signaling. Our microarray data had revealed fewer cardioprotective pathways being modulated in *C. albicans*-infected BcA17 hearts in comparison to similarly infected B6 hearts [17], suggesting a compromised reaction to the inflicted injury. We therefore identified 5 genes that have been implicated in the response to stress and whose expression was higher in *C. albicans*-infected B6 than similarly infected BcA17 hearts and examined their expression in *C. albicans*-infected C5aR^−/−^ mice. These include *Nrp1*, the transcript for neuropilin 1, a non-tyrosine kinase transmembrane molecule, which functions as a co-receptor for VEGFR2. The activation of VEGFR2 signal transduction pathways is crucial for the response to ischemic cardiac injury [30] and Nrp^−/−^ mice are much more susceptible to stress overload than their wild-type littermates [31]. Also important for recovery from pressure overload is calreticulin, since it inhibits the reactivation of the fetal gene program via src down-regulation [32]. Being a Ca^2+^-buffering protein in the endoplasmic reticulum, calreticulin regulation is crucial for cardiac function. *Hspa5* [33] and *Serpinh* [34] are two other transcripts that are inefficiently induced in *C. albicans* infected C5-deficient hearts. The corresponding proteins, HSPA5 (also known as 78 kDa, BiP, GRP78) and HSP47 are also key players in the cardiac response to stress. Indeed, XBP1, a transcription factor induced in response to unfolded protein response (UPR) stress, activates the expression of both HSPA5 and BNP [35]. Overexpression of HSPA5 attenuates GADD45 expression and cardiomyocyte apoptosis caused by proteasome inhibitors [36] and down-regulation of GADD45 is a known survival mechanism [37]. B6 and BALB/c hearts up-regulate *Hspa5* more efficiently than C5-deficient A/J, BcA17 and C5aR^−/−^, and are thus able to prevent the remarkable *Gadd45* increase seen in the absence of functional C5a, supporting the idea of sub optimal stress response in these mouse strains. Finally this phenotype is not restricted to *C. albicans* infection, since C5-deficient A/J, BcA17 and C5aR^−/−^ hearts were also more sensitive to isoproterenol administration as evidenced by higher levels of *Nppa* than C5-sufficient B6 and BALB/c hearts. Our results implicating C5a in cardioprotective responses are consistent with the reported role of C5a in triggering cell survival signaling [38] in the liver and priming quiescent hepatocytes to re-enter the cell cycle during liver regeneration [39], [40], thus raising the exciting possibility that C5 may be a key factor in inducing a similar cell survival stimulus in the heart. These results are also consistent with those of Faulx *et al.* [26] who reported that the A/J heart was more seriously damaged than the B6 heart upon administration of isoproterenol, a trigger of cardiac hypertrophy and apoptosis [41], and b), Hoit *et al.* [42] who reported that the B6 heart was more “athletic” than the A/J heart, displaying physiologic hypertrophy, lower heart rate and greater exercise endurance. Thus the second important conclusion of our study is that in the absence of C5a function, cardiac stress response is compromised. The BcA17 and C5aR^−/−^ mice provide ideal models to identify cardioprotective pathways specific for various forms of cardiac injury.

In conclusion, we have demonstrated that C5-deficiency has a profound effect on cardiac function. Although the normal physiological role of C5 in the context of response to infection has been well established, our results suggest a novel role for C5 in normal cardiac signaling. Our results also suggest that the pharmacological modulation of C5 activity in certain pathological conditions in humans must be approached with caution.
